# Acceptability of broadly neutralizing antibodies (bNAbs) for HIV prevention among vulnerable populations in India: Findings from a qualitative study

**DOI:** 10.1371/journal.pone.0321725

**Published:** 2025-04-21

**Authors:** Joyeeta Mukherjee, Saif ul Hadi, Venkatesan Chakrapani, Shruta Rawat, Aylur Kailasom Srikrishnan, Vaishali Mahendra, Dicky Baruah, Shobha Mohapatra, Usha Gopinath, Alpana Dange, Pratyasha Rath, Pallavi Manchi, Alok Gangaramany, Pritha Aggarwal, Shelly Malhotra, Margaret Keane, Rajat Goyal

**Affiliations:** 1 IAVI, Gurugram, India; 2 Centre for Sexuality and Health Research and Policy (C-SHaRP), Chennai, India; 3 Research Division, The Humsafar Trust, Mumbai, India; 4 Implementation Research Division, Y.R. Gaitonde Centre for AIDS Research and Education (YRGCARE), Chennai, India; 5 The Final Mile, Mumbai, India; 6 Global Access, IAVI, New York, United States of America; Boston University School of Medicine, UNITED STATES OF AMERICA

## Abstract

With newer advances in HIV biomedical research, the prevention toolbox is expanding with potential inclusion of long-acting anti-retrovirals, broadly neutralizing antibodies (bNAbs), and vaccines. While a multitude of prevention options provide diverse choice sets, they may potentially introduce conundrums and trade-offs which influence end-user decisions on acceptability. Thus, to maximize the unique value and uptake of new products, it is critical to understand contextual drivers of choice and relative product positioning. With HIV bNAbs in the pipeline, a qualitative study was conducted to understand end-users’ acceptability. 36 focus-group discussions (n=242) and 57 in-depth interviews were conducted in Chennai, Delhi and Mumbai, with female sex workers (FSWs), men who have sex with men (MSM), people who inject drugs (PWID) and transgender women (TGW) and adolescent girls and young women (AGYW). In addition, 15 simulated behavioral experiments (n=94) were conducted to delve deeper into factors influencing decision-making and potential avenues for intention-action gaps to understand preference construction and reversal pathways. Efficacy, frequency of administration and side-effects were the most important attributes driving acceptability. At least 70% to 90% efficacy was preferred. Arm was the most preferred site of administration (familiarity, maximum privacy), whereas buttocks were preferred by some (better pain tolerance, unhampered mobility). One injection every 3–6 months from community-based facilities was most preferred. Most did not favor self-administration (lack of self-efficacy, adverse events) and voiced confidence in community-friendly professionals. Concerns were raised about potential major side-effects such as interactions with co-morbidities, fertility, pregnancy, gender-affirmation therapy, physical appearance. When end-users were presented with hypothetical product profiles, all preferred the non-antiretroviral (non-ARV) based option which had arm as site of injection and administered every three months by healthcare workers. The preference construction journey revealed positive emotions and rational considerations which favoured bNAbs use but they were contrasted with negative emotions and rationale which hindered acceptability, and the users were faced with multiple conundrums. Some of these conundrums gave rise to potential scenarios of preference reversal.

## 1. Introduction

The last decade has seen a remarkable decrease in AIDS-related deaths due to rapid scale-up of antiretrovirals - both for treatment and prevention. This success in saving lives, however, has not been matched with equal success in reducing new HIV infections. In 2023, around 1.3 million people became newly infected with HIV and an estimated 39.9 million people are still living with HIV globally [1]. India has the second largest HIV epidemic in the world, with 2.5 million people currently living with HIV, and approximately 68,000 new infections in 2023 [2].

Globally a range of behavioral and biomedical HIV prevention interventions and strategies are being implemented. These include promotion of male and female condoms, adoption of clean needles, opioid substitution therapy, voluntary male medical circumcision, and biomedical tools such as HIV pre-exposure prophylaxis (PrEP), and the treatment of people living with HIV to achieve viral suppression. However, there are other prevention options in the pipeline such as vaginal rings, microbicides, long-acting injectable and implantable anti-retrovirals (ARVs), multi-purpose prevention tools (MPTs), broadly neutralizing antibodies (bNAbs), and next generation vaccines – all of which are being considered as potential additions to the prevention toolbox [3]. HIV bNAbs are antibodies that can potentially be used to fight against multiple strains of HIV and have advantages over other biomedical prevention tools such as the ability to target and act against a broad spectrum of viral strains, longer half-life, no risk of development of resistance to ARVs used for treatment, and are comparatively safer with rare cases of adverse side effects [4,5]. The World Health Organization (WHO) has also recently published the preferred product characteristics for monoclonal antibodies for HIV prevention [6] and it highlights that based on population-specific needs, mAbs should have longer-lasting protection, minimal side effects and be delivered as injections, among others. A combination of antibodies that bind to different regions of the HIV envelope would ensure maximal coverage, decrease the risk of virus escape, and maintain viral suppression for extended periods of time. Globally, researchers are working on combinations of bNAbs engineered with extended half-lives and increased potency to ensure lower dosage and lesser frequency that would make the end-product accessible and affordable. Many of these bNAb cocktails are currently in early stages of clinical development across the globe and efforts are also underway in India to identify region-specific bNAb cocktails suited to the epidemiological need of the country.

The HIV epidemic in India is concentrated among key populations like female sex workers (FSWs), men who have sex with men (MSM), people who inject drugs (PWID) and transgender women (TGW). The prevalence ranges from 1%–6% (PWID – 6.3%, TGW – 3.1%, MSM – 2.7%, FSWs – 1.6%) with high inter-state variations [7]. Similarly, significant gender-based gaps persist among adolescent girls and young women (AGYW) with HIV prevention awareness levels at 21.7%, HIV testing at 14%, contraceptive adoption level at 28–46% and intimate partner violence at 22% [8]. The National AIDS Control Organization (NACO) has launched several programs to combat HIV in India, including the five-year program on Youth United for Victory on AIDS (YUVA) that aims to raise awareness among young people [9]; successive National AIDS Control Programmes (NACP); and the targeted intervention programmes that focus on behavior change among high-risk groups [10]. These programs have resulted in significant decrease in HIV infections and AIDS related deaths. However, there is evidence of emerging hotspots and rising pockets of infection across the country [11] indicating the urgent need for biomedical prevention options. In India, most of the biomedical prevention products mentioned above are not yet rolled out as a part of the national policy. Thus, there is a need to understand the acceptability of bNAbs vis-à-vis other prevention options.

**Fig 1:**
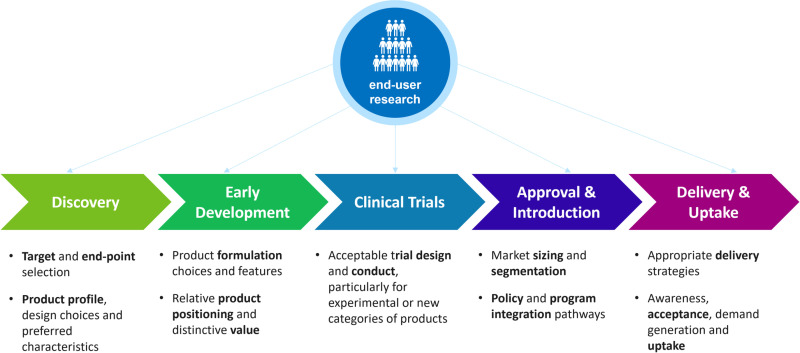
Importance of end-user research at all stages of product development.

While demand estimation exercises often focus on market sizing, the plurality and multi-dimensionality of end-user preferences across populations, geographies and social contexts is often not accounted for. End-user acceptability and preference research play a crucial role in the design, development, evaluation, implementation and uptake of any new product in any geographical setting [12]. They also play a critical role in the development of clinical guidance and policies [Fig 1].

There is a state of constant evolution in the (a) life journeys of **end-users** including their past experiences, trust, knowledge, attitude and perceptions; (b) **prevention product** options including their profiles and characteristics; and (c) contextual **environment** including social norms, structural inequalities, media and misinformation. This gives rise to dynamic preferences which may change based on the conscious and unconscious drivers and real-world conditions. It has been highlighted in multiple studies that a single product will not be preferred by all end-users under all circumstances [13,14]. There is a need to create a toolbox with choice-sets that will cater to the unique needs of the end-users [15]. While the multitude of prevention options provide diverse choice-sets to end-users, they have the potential to introduce conundrums and trade-offs which influence decisions on the uptake of these prevention options in the toolkit [16,17]. These choices, trade-offs, conundrums and the dynamic nature of the preferences often gives rise to “say-do” gaps where the stated preferences of the end-users finally do not translate into observed behaviors at the time of product uptake [18]. Thus, to maximize unique value and uptake of new products in a rapidly evolving HIV prevention landscape, it is critical to understand end-user preferences for various product attributes, contextual drivers of choice, preference construction journeys, key decision pathways, unique needs of various users and relative positioning of different products.

To address these research gaps and needs, the current study aimed to highlight insights on key dimensions of acceptability of bNAbs, and their potential role as prevention products among various key and vulnerable populations including FSWs, MSM, TGW, PWID, and AGYW in India.

## 2. Methods

### 2.1. Study design

The main research questions that the study aimed to answer are:

a. What are the preferred product attributes of bNAbs?
b. What is the relative positioning of bNAbs among other HIV prevention products?
c. What are avenues and preferences where potential “say-do” gaps can potentially occur?

Towards the above, the study was conducted using a generic descriptive-interpretive qualitative research approach [19]. From 11^th^ December 2020–10^th^ November 2021, 37 focus-group discussions (FGDs) (n=242) and 57 in-depth interviews (IDIs) were conducted in Chennai, Delhi and Mumbai, with MSM, TGW, FSWs, PWID and AGYW. To explore diverse perspectives on preferences for product attributes, as well as key behavioral factors influencing adoption and uptake of bNAbs, maximum variation purposive sampling [20] was used to recruit individuals from diverse backgrounds such as those who are engaged in sex work; persons with diverse relationship status (single, co-habiting with male/female partners, heterosexually married) and socio-economic backgrounds; and male and female persons who inject drugs. The participants were recruited through various community-based organizations and non-governmental organizations located in the study sites. Inclusion criteria for MSM, TGW, PWID and FSWs were that they must be at least 18 years of age, self-identified as MSM/TGW/PWID/FSW and willing to provide informed consent. Inclusion criteria for AGYW were that they should be 18–24 years of age and willing to provide informed consent. For FGDs, diverse typologies and subgroups of MSM (sexual role-based identities such as *kothis* [feminine/primarily receptive role], *panthis* [masculine, primarily insertive role] and double-deckers/versatile, and sexual identities such gay and bisexual men), FSWs (brothel/bar-based, street-based, internet/social media-based, home-based), TGW (gharana-based, non-gharana; those in sex work and who are not); PWID (who use different types of injectable drugs) and AGYW across the age group (adolescents 18–19 years and young women 20–24 years) were recruited. In general, about 3–4 FGDs per end-user group are deemed to be sufficient to achieve ‘data saturation’ or ‘theoretical/conceptual saturation’ (no new findings or concepts emerge from the analysis after a particular number of focus groups or interviews) [21]. Thus, to ensure representativeness of all populations of interest in high prevalence pockets in India, it was aimed to have at least 6 FGDs per end-user group (at least 2 FGDs per end-user group per site; for 5 end-user groups across 3 sites = ~30 FGDs). To complement the FGDs, at least 9–10 IDIs per end-user group (at least 3 IDIs per end-user group per site; for 5 end-user groups across 3 sites = ~ 45) were also planned. A total of 37 FGDs and 57 IDIs were conducted to ensure comprehensive coverage till data saturation was achieved.

**Fig 2:**
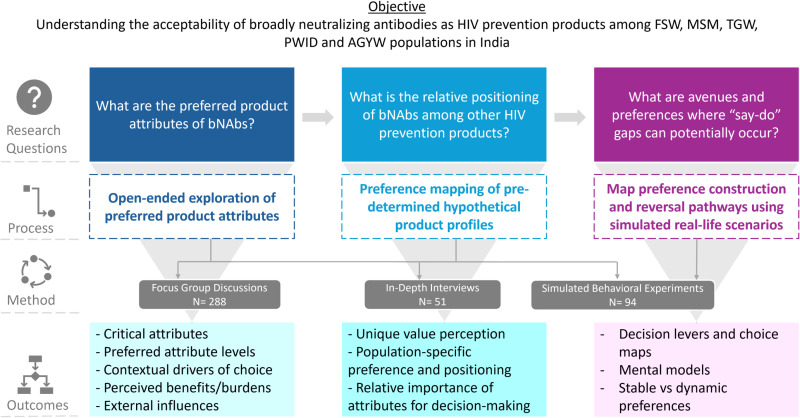
Study overview.

In addition to the FGDs and IDIs, during the same timeframe, 15 simulated behavioral experiments (SBEs) were conducted among all study populations in small batches of 5–6 participants per study population - using the same sampling methodology and inclusion criteria - to delve deeper into the factors influencing decision-making and potential avenues for intention-action gaps by employing interdisciplinary concepts from behavioral economics and decision science to understand preference construction and reversal pathways. The observation of actual human behavior in simulated experiments enables the identification of behavioral deviations from theory, and thus elucidates the underlying interplay of human emotions, motivations, heuristics and biases [22]. The study snapshot of research questions, process and methods followed and intended outcomes are summarized in Fig 2.

The study protocol was approved by the institutional review boards of the Humsafar Trust (HST), Y.R. Gaitonde Centre for AIDS Research and Education (YRGCARE) and the Centre for Sexuality and Health Research and Policy (C-SHaRP). The approved protocol (HST-IRB-50–01/2021; C-SHaRP/0007327/220; YRGCARE-359) was implemented for data collection and analysis. Written informed consent was obtained from all study participants before data collection and the collected data were de-identified during transcription. The de-identified data were used for further analysis

### 2.2. Data collection

Data from FGDs and IDIs were collected using semi-structured interview topic guides (Supporting Information S1, S2, S3 and S4) by trained research staff. SBEs were conducted by partner consultants who went through an immersion phase for alignment with findings from the FGDs and IDIs. The topic guides were translated into native languages (Hindi, Tamil, Telugu or Marathi), back-translated into English and then revised in the original language. All discussions were audio-recorded, transcribed and translated. Each FGD and IDI spanned over 60–90 minutes. All participants received around 500 INR (~6 USD) for their participation.

**Fig 3:**
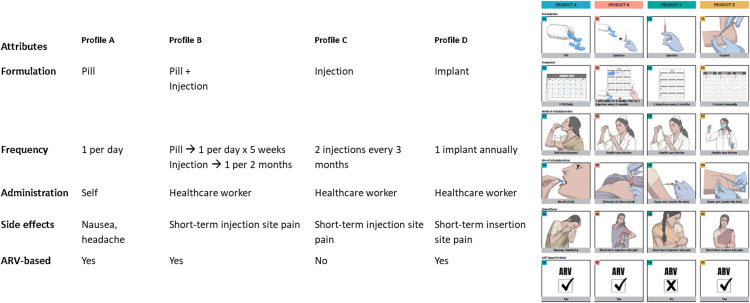
Product profiles presented to end-users for choosing the most preferred and least preferred bundles.

For the FGDs and IDIs, the qualitative enquires involved the following stages:

1. **Open-ended exploration of preferred product attributes:** In this section, preferences for individual product attributes and different levels within that attribute for HIV bNAbs were explored [Fig 2]. This phase aimed to understand which attributes mattered most to the populations and the reasons behind the preferred options. This section also covered the individual, interpersonal and social factors that drove the end-user decision making.
2. **Preferences of pre-determined product profiles**: Towards the end of each FGD and IDI, participants were presented with four hypothetical product profiles comprising five attributes in different combinations (bundles) using IEC tools [Fig 3]. These were modelled after HIV prevention products such as oral PrEP, long-acting injectable PrEP, HIV bNAbs and implantable multi-purpose prevention technology. However, these were not revealed to the participants at the time of data collection but only presented as profiles A, B, C and D. Participants were asked to choose their “most preferred” and “least preferred” profiles and explain the reasons behind their choices. In addition to enabling a deeper understanding of preferences and the unique value perception of bNAbs, this also aided in elucidating the conundrums at play, as well as population-specific preferences and the most critical attributes informing decision-making on product use among prospective end-users [Fig 2].

SBEs were conducted by co-investigators who went through an immersion phase (preliminary analysis of FGD/IDI data) for alignment with findings from the FGDs and IDIs. For SBEs, the focus was on understanding the following:

3. **Preference construction journeys and preference reversal pathways** –SBEs used the insights generated from the FGDs and IDIs to construct a range of decision scenarios which end-users could face in deciding their preferences for a potential prevention product. SBEs then used a game-based research technique (Ethnolab) [18] which included a card-based game play (~ 60 minutes) followed by group discussions (~ 45 minutes). SBEs helped identify decision levers and choice maps, understand mental models and outline stable versus dynamic choices in the context of simulated near real-life scenarios [Fig 2].

### 2.3. Data analysis

**Fig 4:**
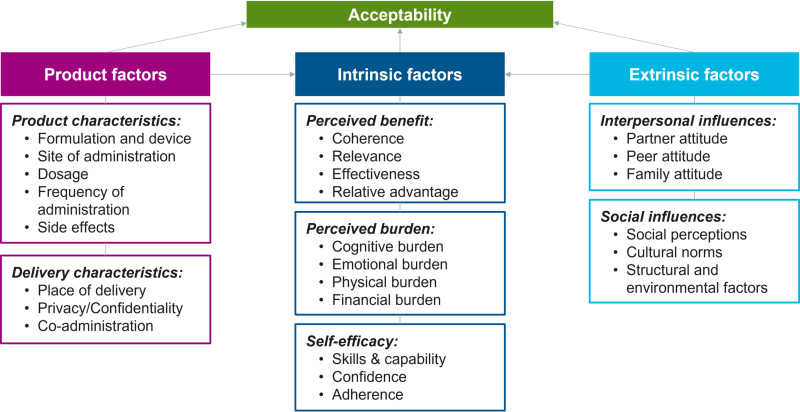
Conceptual framework for acceptability (Reproduced from [ 23]; it was published under the terms of Creative Commons Attribution 4.0 license).

Data were explored through thematic and framework analytic techniques, using the conceptual framework of acceptability [Fig 4] for this study [23] that was adapted from the theoretical framework of acceptability by Sekhon *et al* and Mensch *et al* [24,25]. All transcripts were redacted and uploaded into Dedoose™. First-level codes were identified to create a common *a priori* codebook based on the conceptual framework [Fig 4] and topic guides. Texts corresponding to each of the first-level codes were coded by at least two independent analysts and reviewed by senior investigators. Explanatory/theoretical or etic (researcher-generated) codes were arrived at using a constant comparative method [26].

The SBE results were analysed using the cognitive and emotional appraisal framework [27]. The SBEs were analysed to have an in-depth understanding of: (a) preference construction; and (b) preference reversal. Methods triangulation (FGDs, IDIs and SBE) and researcher triangulation helped in strengthening the validity of the inferences [19].

## 3. Results

### 3.1. Sociodemographic characteristics

A total of 393 people (87 MSM, 71 FSW, 82 TGW, 80 PWID and 73 AGYW) participated in this study across the three sites in FGDs, IDIs and SBEs. In the FGDs (Table 1), the mean age of participants ranged from 20–35 years. Their mean monthly personal income ranged from 3400 INR to 14600 INR (MSM – 10438 INR; TGW-14695 INR; FSW- 5551 INR; PWID – 7220 INR; AGYW – 3481 INR). For MSM, TGW and AGYW, the highest level of education for most participants was secondary and graduate level education; whereas for FSW and PWID the highest level of education for most participants was primary or secondary education (Table 1). MSM identified themselves using sexual role-based identity categories such *kothi (feminine and receptive role) – 43%, panthi (masculine and insertive role) – 11%,* double decker (insertive and receptive/versatile) – 9%, or sexual identity categories such as bisexual – 11%; and gay men – 17%. Majority of the TGW identified themselves as trans women or thirunangai (69%) and Hijra (16%). Among the AGYW, 43% were adolescent girls aged between 18–19 years of age, 52% were young women with age ranging from 20–24 years and 5% were pregnant women. Majority of MSM (84%), TGW (84%); PWID (59%) and AGYW (74%) were unmarried whereas 71% of the FSWs were married. About one-third of MSM, half of the TGW, all of the FSW and none of the PWID reported engaging in sex work. The MSM were majorly involved in jobs with private companies (30%), NGOs (16%) or were students (16%). The FSWs were home-based, network-based and call-based, with their clientele ranged from 20 to 700 clients at the time of this data collection. Most of the TGW were single, with sex work (33%) or begging (20%) as their primary occupation. Majority of the PWID worked as labourer (22%); salesmen (14%), rag picker (10%) and other tertiary work like painter, driver, helper, carpenter, shopkeeper, etc. AGYWs were also majorly involved in private jobs but a significant number of them (48%) were also unemployed. Among all participants, majority of those who revealed their HIV status reported being negative except in FSWs where 5% of the participants reported a positive status.

**Table 1:** Sociodemographic characteristic of the participants in FGDs (n=242).

Characteristics	Focused Group Discussions (FGDs)				
	MSM N=57 (%)	TGW N=51 (%)	FSW N=41 (%)	PWID N=51 (%)	AGYW N=42 (%)
**Mean Age in years (SD)**	28.2 (8.6)	20.9 (6.9)	35.5 (8.3)	28.2 (11.5)	20.4 (2.18)
**Highest level of education**					
Illiterate	0	5 (9.80)	13 (31.71)	1 (1.96)	0
Primary	3 (5.26)	4 (7.84)	14 (34.15)	24 (47.06)	0
Secondary	32 (56.14)	26 (50.98)	12 (29.27)	21 (41.17)	26 (61.9)
Graduate	22 (38.59)	16 (31.37)	2 (4.88)	5 (9.80)	16 (38.1)
Not answered	0	0	0	0	0
**Monthly Personal Income (INR)**					
Mean	10438	14695	5551	7220	3481
**Sexual role-based or sexual identities**					
	Kothi – 23 (40.35) Panthi – 6 (10.53) Double decker - 5 (8.77) Bisexual - 6 (10.53) Gay - 10 (17.54) Not answered - 7 (12.28)	Transwomen/ thirunangai – 35 (68.63) Hijra=8 (15.68) Bisexual=1 (1.96) Gay=1 (1.96) Kothi=1 (1.96) NA=5 (9.80)	Sex Worker – 41 (100)	Injecting drug Users – 51 (100)	Adolescent Girls – 18 (42.86) Young Women – 22 (52.38) Pregnant Women – 2 (4.76)
**Relationship Status**					
Unmarried	48 (84.21)	43 (84.31)	7 (17.07)	30 (58.82)	31 (73.8)
Married	8 (14.04)	0	29 (70.73)	18 (35.29)	11 (26.19)
Widow/Widower	1 (1.75)	0	4 (9.76)	0	0
Separated	0	0	1 (2.44)	3 (5.88)	0
Has a regular partner	0	8 (15.68)	0	0	0
**Main Occupation**					
	Student – 9 (15.79) Self–employed – 1 (1.75) Private job – 17 (29.82) NGO – 9 (15.79) Teacher – 1 (1.75) Unemployed – 12 (21.05) Not Answered – 8 (14.04)	Mangti– 10 (19.61) Pvt Job – 6 (11.76) Sex work – 17 (33.33) Self – employed – 1(1.96) Pan – 1 (1.96) Modelling – 2 (3.92) NGO – 3 (5.88) Unemployed – 1 (1.96) Not Answered – 3 (5.88)	Sex Work – 36 (87.80) Bar Girl – 1 (2.44) Catering – 3 (4.88) Daily wage – 1 (2.44)	AC mechanic – 2(3.92) Labourer – 11(21.56) Painter – 4(7.84) Helper– 4(7.84) Driver –4(7.84) Shopkeeper – 3 (5.88) Carpenter – 1 (1.96) Rag picker – 5 (9.80) Salesman – 7(13.72) Businessman – 1 (1.96) NGO – 5(9.80) Unemployed – 4(7.84)	Student – 8 (19.04) Self–employed – 3 (7.14) Private job – 7 (16.66) NGO – 3 (7.14) Shopkeeper – 1 (2.38) Unemployed – 20 (47.62)
**Involved in Sex work**					
Yes	16 (28.07)	29 (56.86)	41 (100)	0	–
No	18 (31.58)	9 (17.65)	0	51 (100)	–
Not Answered/ Not Asked	23 (40.35)	13 (25.49)	0	0	42 (100)
**HIV Status**					
Negative	27 (47.37)	25 (49.02)	13 (31.70)	27 (52.94)	25 (59.52)
Positive	0	0	2 (4.88)	0	0
Not Tested	0	0	0	0	6 (14.28)
Not answered/ not asked	30 (52.63)	26 (50.98)	26 (63.41)	24 (47.06)	11 (26.19)

For the participants in IDIs (Table 2), the mean age ranged from 21–40 years and their mean monthly personal income ranged from 3300 INR to 16200 INR. The highest level of education for most participants was secondary and graduate level education. 50% of the MSM identified themselves as *kothi* and 33% as gay men; 69% of TGW were trans women or *thirunangai* and 8% were Hijra. Among the AGYW, 27% were adolescent girls aged between 18–19 years of age, 45% were young women with age ranging from 20–24 years, 18% were pregnant women and 9% were mothers of infants aged between 4 months to 3 years. All of the TGW were single whereas for the other populations there was a fair balance of married and unmarried participants. Similar trends were followed as the FGD participants for reported engagement in sex work. The MSM were majorly involved in jobs with private companies (25%) or NGOs (42%). The FSWs were all involved in commercial sex work and most of the TGW were had sex work (23%) or begging (31%) as their primary occupation. The PWIDs included in the IDIs were majorly social workers and most of the AGYWs were unemployed. Among all participants, majority of those who revealed their HIV status reported being negative except in FSWs where 1 of the participants reported a positive status.

**Table 2:** Sociodemographic characteristic of the participants in IDIs (n=57).

Characteristics	In-Depth Interviews (IDIs)				
	MSM N=12 (%)	TGW N=13 (%)	FSW N=12 (%)	PWID N=9 (%)	AGYW N=11 (%)
**Mean Age in years (SD)**	35 (10.5)	31.3 (9.8)	40.1 (6.4)	27.5 (11.3)	21.1 (2.2)
**Highest level of education**					
Illiterate	0	1 (7.7)	1 (8.3)	0	0
Primary	0	0	3 (25.0)	1 (11.1)	0
Secondary	7 (58.3)	8 (61.5)	8 (66.7)	5 (41.7)	4 (36.4)
Graduate	5 (41.7)	4 (30.8)	0	3 (25.0)	6 (54.5)
Not answered	0	0	0	0	1 (9.1)
**Monthly Personal Income (INR)**					
Mean	16220	14083	15250	9928	3300
**Sexual role-based or sexual identities**					
	Kothi – 6 (50.0) Panthi – 0 Double decker - 0 Bisexual – 1 (8.3) Gay – 4 (33.3) Not answered – 1 (8.3)	Transwomen/ thirunangai – 9 (69.2) Hijra=1 (7.7) NA=3 (23.1)	Sex Worker – 41 (100)	Injecting drug Users – 51 (100)	Adolescent Girls – 3 (27.3) Young Women – 5 (45.5) Pregnant Women – 2 (18.2) Mother of infants – 1 (9.1)
**Relationship Status**					
Unmarried	9 (75.0)	13 (100)	3 (25.0)	3 (25.0)	7 (63.6)
Married	3 (25.0)	0	8 (66.7)	6 (50.0)	4 (36.4)
Widow/Widower	0	0	0	0	0
Separated	0	0	1 (8.3)	0	0
Has a regular partner	0	0	0	0	0
**Main Occupation**					
	Private job – 3 (25.0) NGO – 5 (41.6) Unemployed – 2 (16.7) Sex work – 2 (16.7)	Mangti– 4 (30.8) Pvt Job – 1 (7.7) Sex work – 3 (23.1) Jogathi – 1 (7.7) NGO – 4 (30.8)	Sex Work – 9 (75.0) Bar Girl – 3 (25.0)	Social worker – 4 (33.3) NGO – 2 (16.7) Pvt job– 1 (8.3) Unemployed – 2 (16.7)	Student – 1 (9.1) Self–employed – 1 (9.1) Private job – 1 (9.1) Teacher – 1 (9.1) House help – 1 (9.1) Unemployed – 6 (54.5)
**Involved in Sex work**					
Yes	4 (33.3)	6 (46.2)	12 (100)	0	0
No	4 (33.3)	3 (23.1)	0	0	0
Not Answered/ Not Asked	4 (33.3)	4 (30.8)	0	9 (75.0)	11 (100)
**HIV Status**					
Negative	6 (50.0)	5 (38.5)	2 (16.7)	0	8 (72.7)
Positive	0	0	1 (8.3)	0	0
Not Tested	0	0	0	0	0
Not answered/ not asked	6 (50.0)	8 (61.5)	9 (75.0)	9 (75.0)	3 (27.3)

For the SBE participants, mean age ranged from 23–32 years and other demographic trends were similar to the participants in the FGDs and IDIs (Table 3).

**Table 3:** Sociodemographic characteristic of the participants in SBEs (n=94).

Characteristics	Simulated Behavioral Experiments (SBEs)				
	MSM N=18 (%)	TGW N=18 (%)	FSW N=18 (%)	PWID N= 20 (%)	AGYW N=20 (%)
**Mean Age in years (SD)**	26.1 (4.5)	26.2 (5.5)	29.7 (6.7)	32.2 (7.2)	23.9 (2.2)
**Highest level of education**					
Illiterate	0	0	0	3 (15.0)	0
Primary	0	0	1 (5.5)	1 (5.0)	0
Secondary	7 (38.8)	6 (33.3)	16 (88.9)	6 (30.0)	7 (35.0)
Graduate	4 (22.2)	6 (33.3)	1 (5.5)	0	7 (35.0)
Not answered	7 (38.8)	6 (33.3)	0	10 (50.0)	6 (30.0)
**Sexual role–based or sexual identities**					
	Kothi – 9 (50) Panthi – 1 (5.5) Double decker – 0 Bisexual – 8 (44.4) Gay – 0 Not answered – 0	Transwomen/ thirunangai – 7 (38.8) Hijra=5 (27.8) NA=6 (33.3)	Sex Worker – 18 (100)	Injecting drug Users – 20 (100)	Adolescent Girls – 3 (15.0) Young Women – 13 (65.0) Pregnant Women – 1 (5.0) Mother of infants – 3 (15.0)
**Relationship Status**					
Unmarried	12 (66.7)	15 (83.3)	3 (16.7)	11 (55.0)	13 (65.0)
Married	5 (27.8)	0	11 (61.1)	8 (40.0)	6 (30.0)
Widow/Widower	1 (5.5)	0	2 (11.1)	0	0
Separated	0	0	2 (11.1)	0	0
Has a regular partner	0	3 (16.7)	0	1 (5.0)	1 (5.0)
**Main Occupation**					
	Student – 2 (11.1) Private job – 8 (44.4) NGO – 4 (22.2) Self employed – 1 (5.5) Delivery guy – 1 (5.5) Shopkeeper – 1 (5.5) Sex work – 1 (5.5)	Mangti– 5 (27.8) Self employed – 1 (5.5) Sex work – 7 (38.8) Jogathi – 2 (11.1) NGO – 2 (11.1) Unemployed – 1 (5.5)	Sex Work – 16 (88.9) Catering – 1 (5.5) Nurse – 1 (5.5)	Migrant worker – 3 (15.0) Driver – 1 (5.0) Salesman – 2 (10.0) Pvt job– 1 (5.0) Business – 1 (5.0) Labourer – 4 (20.0) Mechanic – 1 (5.0) Unemployed – 4 (20.0) Not answered – 2 (10.0)	Student – 10 (50.0) Private job – 3 (15.0) Teacher – 2 (10.0) Unemployed – 1 (5.0) Not answered – 4 (20.0)
**Involved in Sex work**					
Yes	7 (38.8)	12 (66.7)	18 (100)	0	0
No	11 (61.1)	6 (33.3)	0	0	0
Not Answered/ Not Asked	0	0	0	20 (100)	20 (100)

### 3.2. Open–ended exploration of preferred product attributes for HIV bNAbs

**Fig 5:**
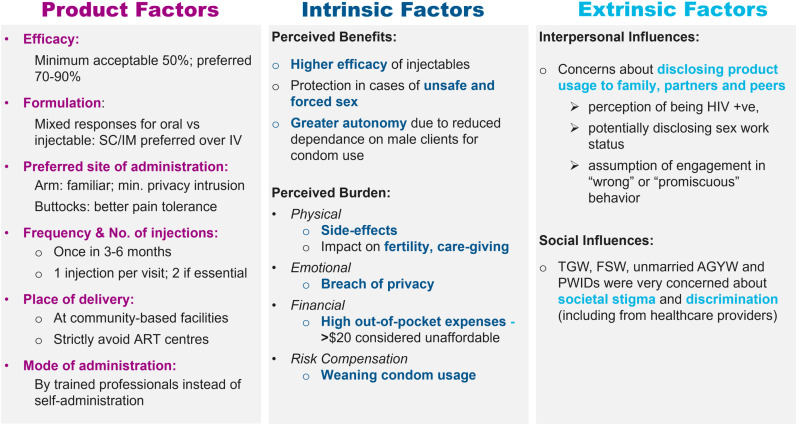
Factors affecting end–user acceptability of bNAbs as prevention products.

The open–ended exploration focused on individual preferences for: (a) product factors like efficacy, formulation, site of administration, frequency and number of injections, place of delivery and mode of administration; (b) Intrinsic factors like perceived benefits and perceived burden; and (c) Extrinsic factors like interpersonal and social influences. The findings have been summarized in Fig 5 based on the conceptual framework of acceptability.

#### 3.2.1 Product based factors.

*Efficacy*

Participants reported efficacy as the most important criterion for driving decisions on product uptake. Most participants had a fair understanding of the term “efficacy” and associated it with “level of protection” and “safety”. Minimum acceptable efficacy voiced by end–users was 50% and products with efficacy ranging between 70%–90% had the highest acceptance.

*“It should be 70–90%. The main reason is that when we are using any product and consuming it then we* ***want it to provide us safety****.” (MSM, IDI–2, Delhi)*

*“It should be between 70 and 90%. Because at–least this percentage will ensure that the product provides* ***a reasonable level of protection****.” (PWID, FGD, Chennai)*

*Formulation*

There were mixed responses for oral and injectable formulations.

Respondents in most instances indicated their willingness to use injectable formulations if that was the only option towards providing more effective health outcomes. PWID, MSM, and TGW were comfortable with injectable formulations as they perceived injections to have higher efficacy and better adherence than pills.

*“I think injection is better because you might forget to take pills but* ***injection will get dissolved in your body at once and will be more effective****. Medicines start affecting late as it gets dissolved late.” (TGW, FGD, Delhi)*

*“Tablet is quite difficult. Because if we are taking it in* ***injection form then it will be one time thing and it’s an easy method****. But if you have to take tablets every day then it is quite difficult. You know how our life becomes busy sometimes so we forget to take it.” (MSM, IDI, Mumbai)*

FSW and AGYW tended to favor pill–based formulations since they perceived pills to provide greater autonomy of use and convenient and easy to access.

*“In tablet, we could take it anywhere no would ask anything, we just take the tablets with us in our purse.” (FSW, FGD, Delhi)*

*“Because it is easy to eat, during travelling it is easy to carry, it would be easy with any situation, when you will be out of station you can have it with you.” (AGYW, IDI, Delhi)*

PWID was the only group that preferred intravenous (IV) infusion since they were familiar with the process. None of the other end–users preferred IV–based infusion as it would take longer, be more painful and need hospital or clinic set up. They preferred subcutaneous or intramuscular mode and perceived pain quotient was the driver of this choice.

*“Yes, I will like it because while using drugs, we inject in veins only.” (PWID, FGD–1, Delhi)*

*Site of administration*

Arm was the most preferred site of administration because of familiarity with the process and minimal intrusion in privacy. Buttocks were preferred by some for better pain tolerance and unhampered physical mobility, but this was often not the first choice because of privacy concerns.

*“It would be comfortable to take injection on arms. Secondly, if we take it on our butt then we need to remove our pant. So, arm would be better option.” (MSM, IDI–1, Delhi)*

*“If it’s on the butt, it’s not painful and we can work easily. Butt pain goes easily, arm pain is there for long.” (MSM, FGD–2, Delhi)*

*Frequency and number of injections*

Participants were comfortable with a frequency of once every 3–6 months as this coincided with their HIV testing schedules. Some were willing to accept a higher frequency of once every month while others felt that any frequency more than once every 3 months would be inconvenient.

*“I feel 6 months is a long period, 1 month is very short, so 3 months is fine. Even HIV test is done once in 3 months.” (MSM, FGD, Delhi)*

*“As most of us are working people so it is not feasible to go every month, maybe once in three months or once in six months we can go.” (AGYW, FGD–2, Chennai)*

Mostly all end–users preferred one injection per visit and voiced that anything more would act as a barrier to product uptake. Some were willing to accept two injections per visit in case it was essential for adequate efficacy and provided it was administered in regions which did not impact daily functioning due to resulting pain.

*“I will take 1 injection per visit, whether that is monthly or after 3 months. Needle gives pain, so we shall prefer 1 injection only.” (MSM, FGD–2, Delhi)*

*“Yes… to prevent HIV, if they ask to put two injections, I will follow the advice given by doctor” (AGYW, IDI–2, Chennai)*

*“It’s like if one is not sufficient then we can get one more injection.” (TGW, IDI–2, Delhi)*

*Place of delivery*

Most end–users preferred accessing bNAbs at community–based facilities as they felt that the settings are more friendly, trustworthy and accepting. Some respondents (MSM, AGYW) preferred public hospital–based settings to avoid the related stigma (both perceived and enacted) and “spotlight” on communities. End–users asserted that they would avoid accessing bNAbs at ART centres due to the fear of being labelled as “HIV positive”. Pharmacies and private clinics were mostly not accepted as they might give rise to potential misuse/overuse of the product and due to the high out–of–pocket expenses.

*“NGO is best for me because they help us more, talk to us politely, keep everything confidential.” (FSW, IDI, Delhi)*

*“People don’t want to go ART centre because other people may see them taking it and all those things are there. That is where these CBOs and NGOs help.” (PWID, FGD, Chennai)*

*Self– administration, assisted administration and co–administration*

Participants did not prefer self–administering bNAbs due to lack of skills/training and self– confidence to use the product. They felt that they did not have the required expertise and feared adverse events from incorrect dosage and the inability to deal with these events. Stigma at family level and the absence of refrigerators at home were other deterrents to self–use.

*“Until a person does not know how to self–administer it should not be taken at home. Because they won’t know whether it goes into their blood or whether it is working on their body or not. There might be very bad effects also.” (MSM, IDI–1, Mumbai)*

End–users indicated preference for administration by trained professionals like community–friendly doctors, nurses and trusted, trained NGO workers. Young women, particularly pregnant women and nursing mothers preferred receiving the product from government authorized settings by certified doctors.

Only PWID were very confident of self–administration because of their familiarity with the process. However, they were concerned about the need for refrigeration since most of them did not have access to it. PWID were very accepting of co–administration with opioid substitution therapy services.

#### 3.2.2 Intrinsic factors.

Intrinsic factors are perceptions or motivations that influence the end–users’ decision–making process. The key intrinsic factors that played a role in the end–user acceptability of bNAbs as prevention products are perceived benefits and perceived burden.

*Perceived benefits*

Most end–users perceived injectable prevention products to have higher efficacy than a pill and hence they felt that bNAbs would provide them with a higher level of safety since they are most likely going to be delivered as injections. They also felt that bNAbs would be particularly useful to provide protection in instances of unsafe and forced sex where condom usage is compromised. Since an injectable formulation of bNAbs would reduce the dependency on male clients for condom use, FSWs and TGW felt that this product would offer them greater autonomy and independence in their daily lifestyle.

*Perceived burden*

End–users detailed many physical, emotional and financial concerns that influenced their preferences.

*Physical Burden* – Most participants had concerns around potential major side–effects but they were willing to deal with minor ones. Major side–effects included cross–reactions with other ongoing medications, long–term organ damage, erectile dysfunction, severe impact on menstrual cycle, fertility, pregnancy, harm to foetus and physical appearance/beauty due to rashes. Young women, particularly, mothers of infants were concerned about the impact on their well–being and in turn their inability to attend to infants. Any side effect which can lead to loss of work hours was not considered to be acceptable and could lead to non–adherence of the product.

*“Mild fever for two days is okay. Arm pain and body ache is okay. Since we will recover from that in couple of days. We do not want any long–term diseases from this effect” (PWID, FGD, Chennai)*

Regarding administration of bNAbs to infants, mothers felt that the infants should at least be few months to few years old till they are strong enough to take the injection. However, if the mother is living with HIV, they were in favour of administering the bNAbs to the infant at birth.

*“Should wait till child is 4–5 years old and strong enough to take such an injection.” (AGYW, IDI–2, Delhi)*

*Emotional Burden* – Participants wanted to conceal their recurrent visits to public health centres or clinics from family, partner or peers. The fear of being perceived as living with HIV was also a major factor that contributed to their emotional dilemma.

*Financial Burden* – All end–users preferred to have the product free of cost through the national programme. In the absence of this integration, they were concerned about potentially high out–of–pocket expenses. Willingness to pay ranged from INR 1000 to INR 1500 annually for most end–users. It was lowest for FSWs and ranged from 200 to1000 INR.

*Risk Compensation –* A few participants from across the study groups expressed concerns about decrease in condom use among users of bNAbs. For example, although sex workers are known to forego condom use for extra money from clients, condomless sex was suspected to increase among sex workers on bNAbs*.*

#### 3.2.3 Extrinsic factors.

Extrinsic factors are external drivers that influence a person’s decision. Some of the extrinsic factors that played a significant role in end–users’ decision to use bNAbs are social influences and interpersonal factors.

Most end–users from key populations were concerned about accidental disclosure of their bNAb use to family, partners and peers for fear of being perceived as HIV positive or potential outing of their engagement in sex work. AGYW feared negative family reaction and an assumption of being involved in “wrong” behaviour, being promiscuous, and lacking moral values.

TGW, FSWs, unmarried AGYW and PWIDs were very concerned about societal stigma and differential attitude and discrimination even from healthcare providers. MSM and married AGYW did not voice such concerns and recommended a more generalized approach for accessibility of any prevention product.

### 3.3. Preferences of pre–determined product profiles

**Fig 6:**
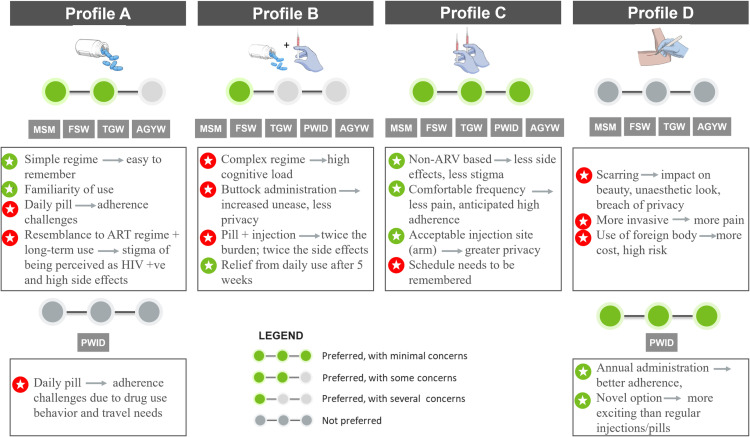
Preference, acceptability drivers and voiced concerns for product bundles by end–users.

When end–users were presented with hypothetical product profiles A, B, C and D [Fig 3], all populations chose profile C to be their most preferred option [Fig 6] as this product was non–ARV based and hence no associated stigma of being considered as living with HIV and lesser side–effects. End–users were comfortable with the frequency of every three months and associated this with tolerable pain and high adherence. They were also accepting of the arm as the site of injection as it provided greater privacy than the buttock administration. However, they did highlight that for this profile, the schedule of injections needs to be remembered and hence, was a minimal concern

All end–users (except PWID) did not prefer profile D [Fig 6] as this was perceived to be highly invasive with the use of a foreign body and hence more painful and very expensive. They also feared potential visible scarring which would have a negative aesthetic impact and breach their privacy.

Some of the PWID, however, chose profile D as their most preferred option along with profile C. They saw appeal in annual administration as it would lead to the best adherence. PWIDs being regular users of injections because of their drug use, the implants provided them with a more novel and exciting option, and they generally reported a higher pain tolerance.

Profile A was less preferred by end–users [Fig 6] primarily because of concerns such as the intensive daily frequency, adherence challenges, resemblance to the ART regimen, associated stigma and side effects due to long–term use of an ARV–based product. However, the familiarity of pill use and simple regime were positive attributes in this profile. Profile A was not preferred by PWID due to the daily adherence challenges which was magnified by their drug use and travel needs.

Preference for profile B was limited among end–users and several concerns were voiced [Fig 6]. The complex regimen was perceived to lead to high cognitive burden. Since it included both pills and injections, end–users felt that they would have to bear the dual burden that came with both forms of administration. Moreover, buttock administration was a cause of discomfort because of the breach of privacy.

### 3.4. Preference construction journey

**Fig 7:**
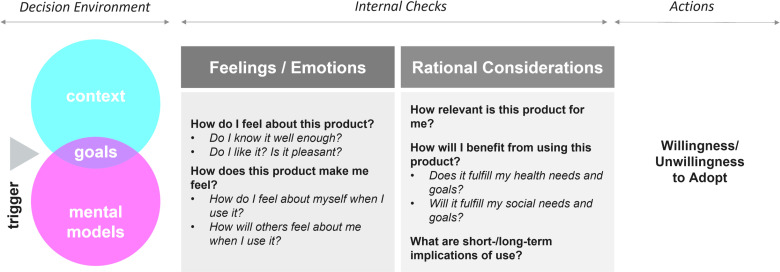
The decision–making framework towards understanding preference construction journey.

The preference construction journey can be broken down into three segments – (a) The trigger and the decision environment; (b) the internal checks and (c) the final actions [Fig 7].

#### 3.4.1. Trigger and decision environment.

The trigger is an event which necessitates the end–user to make a decision. In the current study, the trigger was the potential availability of a new HIV prevention product – bNAbs and the decision or action to finally arrive at was the willingness or unwillingness to use it. Preferences are always context sensitive, influenced by the personal goals of the individual and also impacted by pre–existing mental models [28]. Thus, it is key to understand the complex decision environment in which the preferences were constructed.

A mental model is an explanation of an individual’s thought process and an intuitive perception about his/her own acts and their consequences. Pills were the most common prevention products that end–users were aware of and injections as a mode of administration was familiar to all of the participants. The study indicated the prevalence of particular mentals models around the comparative value and utility of pills (or ARVs in some cases) and injectables arising out of their experience of use and/or contextual knowledge which played a significant role in shaping their final choices. Some of the emerging mental models were around intrinsic pleasantness, efficacy, access, privacy, side effects, burden of use and associated stigma [Table 4].

**Table 4:** Mental Models associated with pills and injectables.

Mental Models: Pills vs Injectables	
**Intrinsic pleasantness:**	pills are large and difficult to swallow; injectables are quick but painful and invasive
**Efficacy:**	pills are slower in effect, have lower efficacy; injectables have better efficacy, work faster than pills
**Access:**	pills are readily available; need to stand in line for injectables which leads to opportunity loss injectables are prone to stock–outs
**Privacy:**	pills are a privacy risk due to home storage; injectables offer privacy; administering on butt is invasive
**Side effects:**	pills have higher side effects due to long–term, daily use; injectables have fewer doses which indicate less side effects
**Burden of use:**	pills are habit–forming; easy routine dose; injectables are not addictive, but difficult to remember doses
**Stigma:**	ARVs are strongly associated with HIV rumors; risk of negative impact on livelihood, social status and client loss

The above mental models, together with the specific context in which the end–user is placed, gave rise to goals around product uptake or use. The major goals which drove the decision pathway were (a) Effort Reduction (b) Control Maximization (c) Privacy Maximization (d) Stigma Avoidance (e) Minimal Pain or Discomfort and (f) Minimal Disruption to Life.

#### 3.4.2. Internal checks and resulting conundrums.

**Fig 8:**
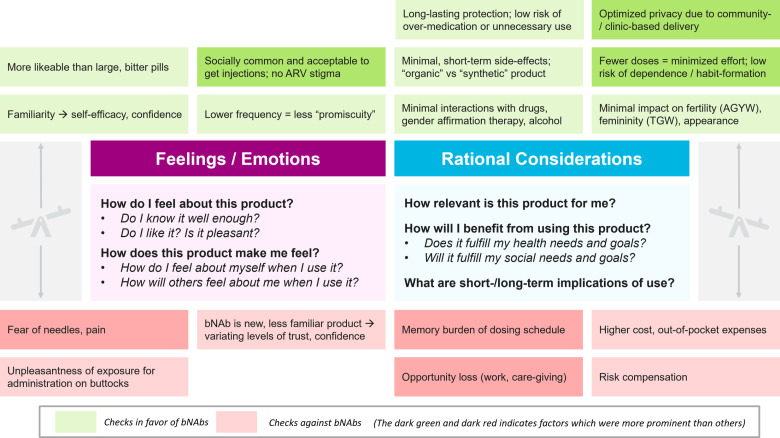
The internal checks that diverse end–user populations consider when deciding the acceptability of bNAbs.

In order to elaborate the preference construction journey, it was imperative to understand the internal checks which the end–user engaged in while working towards a decision/action. These included (a) feelings or emotions about how the individual feels about the product and how the product makes the individual feel; and (b) rational considerations about how relevant the product is, the benefits of using the product and the short–term and long–term implications of use [Fig 8]. All of these taken together enabled the end–user to arrive at a final decision.

Some of the intuitive feelings and emotions which worked in favour of the bNAbs [Fig 8] were high familiarity and likeability, low implied promiscuity and social acceptance. However, the fear of needles and associated pain, the unpleasantness of buttock exposure and the low confidence and trust due to the newness of the product worked against the bNAbs.

The internal checks also encompassed rational considerations around product relevance, product benefit or burden and use implications for the end–user. These spanned across health and social considerations. The rational considerations which worked in favour of bNAbs use [Fig 8] were high perceived efficacy, minimal short–term side effects since bNAbs were non–ARV based products and derived from the human body, optimized privacy and low risk of habit formation. However, the cognitive burden of dosing schedule, the opportunity loss due to side effects, potential weaning condom use, and high out–of–pocket expenses were considered to be barriers to bNAb use.

Thus, there were positive emotions which favoured bNAbs use but they were contrasted with negative emotions which hindered acceptability, and the user was faced with an emotional conundrum. For example, even though injections are a familiar mode of administration, but the fear associated with needles did not make the choice very obvious. Similarly, there were rational considerations which either favoured or hindered bNAb use and the resulting dilemma was noteworthy. For example, even though bNAbs had fewer doses and lesser side effects, if they caused huge loss of work–hours because of long lines at places of delivery, they may not be favoured and there would be adherence issues. Lastly, the emotional versus rational conundrum also arose in cases where there was (a) emotional benefit but rational hindrance (e.g., socially acceptable as it is non–ARV based but high out–of–pocket expense); or (b) rational benefit but emotional hindrance (e.g., fewer doses and less effort but variable trust due to newness of the product). Some of these conundrums gave rise to scenarios of preference reversal.

### 3.5. Potential preference reversal pathways

**Fig 9:**
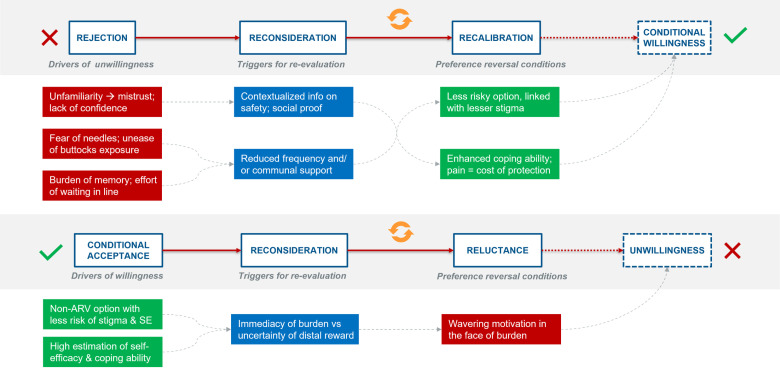
Preference reversal pathways.

A preference reversal usually occurs in two scenarios [Fig 9]:

Scenario 1: When a person is unwilling to adopt a specific product but goes through a process of re–consideration and re–evaluation due to certain changes or/ triggers either in the product itself or in the surrounding context. This leads to a re–calibration of their decisions and a state of conditional willingness for product usage is reached.

Scenario 2: When a person is willing to use a specific product because of certain key attributes but goes through a process of re–consideration and re–evaluation in the face of other burdens of use. This leads to wavering motivation and cropping reluctance and a state of unwillingness for product usage is reached.

In case of bNAbs, preference reversal pathways for both the above scenarios were traced through the study.

Scenario 1: The reasons why end–users were **unwilling** to use bNAbs, which were prone to reversal, were (a) unfamiliarity with the new product and thus arising mistrust and lack of confidence in the product; (b) fear of needles and unease of buttocks exposure; (c) burden of memory and effort of travelling and waiting in line. However, if they were offered certain triggers for re–evaluation, it would allow them to reconsider their decision. The two most important triggers were:

- Provision of extensive contextualized information on product safety and social proof either from trial experience or acceptance of this product in other regions among other populations. This would help address the concern of unfamiliarity, mistrust and lack of confidence in the product.
- Reduced frequency of the product from once in three months to either once in six months or annually. This would allow for better coping ability and reduce the burden of fear, pain, memory, and cost that is associated with more frequent injections.

Scenario 2: The reasons why end–users were **willing** to use bNAbs, which were prone to reversal, were: (a) non–ARV based options with minimal side effects and no associated stigma; (b) high estimation of pain tolerance and coping ability with side effects and societal influences. However, in the face of the immediate burdens (fear of needles, injection site pain, need to remember dates, travel and product cost) and the uncertainty of distal rewards (protection against the risk of HIV), there is wavering motivation and reluctance to adhere to the product.

Thus, frequency of administration was a key factor that played a role in the preference reversal pathway.

## 4. Discussion

The current study highlighted the factors driving the acceptability of bNAbs among five vulnerable populations across three cities in India. To the best of the authors’ knowledge, this is the first study from India that explored community perspectives on this novel HIV prevention option. Globally, several bNAbs are in early phases of development and it was timely to conduct end–user studies to gather their preferences to inform product development. The data generated through this study will aid in defining the relative value of bNAbs in the Indian context, shaping key developmental decisions and ensuring timely evidence–based access pathways.

Across end–users, there was low awareness of bNAbs as a potential prevention product. Most participants reported relying on condoms for protection against HIV transmission. Except for MSM, other study groups lacked knowledge or had never used PEP and PrEP as prevention products – highlighting the need to push for greater awareness and access to existing effective prevention products or those in pipeline. After having received information about bNAbs, participants were open to newer prevention tools and voiced broad acceptability and inclination for bNAbs. Efficacy was a major decision–driver and the minimum acceptable efficacy was 50%. The study recorded mixed preferences for oral and injectable formulations with pills being preferred for ease of use and access whereas injections were perceived to be more efficacious and offered better privacy. For injections, subcutaneous or intramuscular were preferred over intravenous administration and arms and buttocks were preferred sites of administration. End–users preferred taking one injection every 3–6 months delivered through trained healthcare professionals at community–based facilities. They highlighted concerns about potential major side–effects from use of bNAbs, high out–of–pocket expenses, and weaning condom use. However, the attributes that mattered most to the end–users were: (a) efficacy; (b) frequency of administration; and (c) side effects.

The study also provided insights about the unique value proposition of bNAbs as prevention products. It was perceived to be advantageous over other currently available prevention options like PrEP due to: (a) enhanced usability and convenience due to lesser frequency of administration, simplified dosing schedule, and consequent reduction in burden of use (physical, emotional and cognitive); (b) minimize stigma and association with HIV infection, as well as perceptions of fewer side effects due to non–ARV based formulation; and (c) increased privacy due to administration at community–based settings or public healthcare centers.

The findings from this study are consistent with end–user insights generated for other prevention products such as PrEP, long–acting injectables, MPTs, vaginal rings [12,29–31]. Efficacy, formulation (pills, injection, implant, rings, gels), frequency of administration, were strong determinants of choice [12,29,31,32]. Other product attributes which influenced the decision–making of end–users were familiarity, discreetness, side–effects, additional protection from pregnancy and sexually transmitted diseases [12,33,34]. For the majority of these prevention products, end–users described daily dosing schedule as burdensome and preferred a frequency ranging from one month to one year [31]. Preferences were also noted for products that did not interfere with fertility, menstrual cycle, sexual pleasure and long–term co–morbidities [31], same as noted in the current study for bNAbs.

Thus, improving the overall experience of use of the product was critical for end–user acceptability. The findings from the current study implied that as bNAbs move along in the development pathway, efforts should be made to (a) reduce the number of injections and associated pain factor by using smaller needle dimensions (length, width, depth of insertion and volume); (b) reduce side effects to enhance daily functionality post administration and minimizing aesthetic impact; (c) ensure no drug–drug interactions occur (for TGW with female hormones and for PWID with injecting drugs); (d) ensure delivery at general clinics/ public healthcare centres/ community–based settings and not at ART centres; and, (e) facilitate administration through trained professionals like healthcare providers, trained nurses and CBO workers to provide trust and reliability to the end–users. Further, there is a need to generate more awareness among the end–users since this is a relatively newer class of prevention products. Literacy materials on bNAbs should be made available in lay and local languages or other interactive forms to address concerns around adherence, effectiveness, drug interactions, side effects. Behavior change communication campaigns (using various physical, virtual and digital communication strategies) to spread awareness about bNAbs should be conceptualized and implemented. Information about bNAbs should be disseminated widely among the general public instead of only among key populations to avoid stigma and create a positive perception around bNAbs and its users.

Since the conduct of this study, there have been many developments in the HIV prevention product landscape, with the most recent one being the success of lenacapavir twice yearly subcutaneous injections in reducing the HIV incidence [35]. However, the findings from this study are relevant and important even in the current context. When this study was being designed, one of the hypothetical product profiles (Profile B) was modelled after the long–acting injectable PrEP CAB–LA which was the most promising long–acting PrEP option in the pipeline at that time and the findings take into account end–user preferences vis–à–vis the long–acting PrEP option as well. With further improvements, better options like lenacapavir are now on the horizon. However, the current study findings suggest that one of the major drivers for bNAb acceptability among end–users is that it is a non–ARV based product and hence will not be associated with the stigma of being HIV positive. They also perceived higher side effects with ARV–based options and favored the non–ARV–based bNAbs. In addition to all end–users, this was particularly noted among pregnant women and mothers of infants who were concerned about the adverse effects of ARV–based options to the foetus and/or newborn infants.

There are some limitations of the current study. First, the findings from this study cannot be generalized in a statistical sense as participants were recruited via purposive sampling. However, to ensure transferability (applicability of findings to similar contexts/settings), FGDs and interviews were conducted across diverse settings in various cities. Second, it is possible that differences in questioning and probing styles might have led to limited exploration of certain areas of interest. All interviewers received rigorous training on conducting FGDs and IDIs and practiced mock sessions with feedback, both of which might have improved the quality of follow–up and probe questions. Further, the variations in the questioning/moderating styles of the interviewers might have also contributed to rich diversity in the kind of responses that were obtained from the participants.

## 5. Conclusions

The findings of this study emphasized that different populations may have different preferences for combination of product attributes and such choices may be driven by varied lived experiences. While bNAbs were acceptable as prevention products to all populations, with the constantly evolving prevention toolbox, clear articulation of the unique value proposition and positioning of bNAbs which takes into account the segmented preferences of all potential end–users will be extremely important for acceptability and uptake. Product developers should focus on enhancing ease of use, reducing cognitive burden and improving overall experience with the product. It is important to note that reducing the frequency of injections, increasing the efficacy of the product, and minimizing the side effects are important factors that will have a bearing on the end–user acceptability. User–friendly, simple communications around product use, storage and value proposition should be developed. Delivery outlets should include trusted community–based settings. Product developers should also be mindful of the unique needs of diverse end–users and the product relevance for diverse communities while designing the product and defining the unique value proposition. The personal goals, mental constructs and internal checks often lead end–users to make certain choices and trade–offs which may not be stable under all circumstances. Thus, it is important to identify the stable factors and be cognizant of the dynamic preferences and the core drivers of these choices to outline demand generation and implementation strategies.

## Supporting information

S1 File Topic guide for focus group discussions with FSWs, MSM, PWID, TGW. (DOCX)

S2 File Topic guide for focus group discussions with AGYW. (DOCX)

S3 File Topic guide for in–depth interviews with FSWs, MSM, PWID, TGW. (DOCX)

S4 File Topic guide for in–depth interviews with AGYW. (DOCX)
