# Supplementary material for: Acceptability of broadly neutralizing antibodies (bNAbs) for HIV prevention among vulnerable populations in India: Findings from a qualitative study
Source: PLoS One. 2025 Apr 21;20(4):e0321725. doi: 10.1371/journal.pone.0321725 (PMC12011248; doi:10.1371/journal.pone.0321725)
Supplement: S1 File — (DOCX) [file pone.0321725.s001.docx]

**Acceptability and feasibility of broadly neutralizing antibodies as HIV prevention products in India: Exploring the perspectives of potential end-users, service providers and policymakers**

*Topic Guide – Focus Group Discussions – End-Users (FSWs, MSM, PWID, TGW)*

***Instructions for Facilitator:***

- *Greet and check-in participants.*
- *Introduce yourself and explain your role.*
- *Obtain informed consent from each participant before they join the group (unless the note-taker has this responsibility).*
- *Those in italics are the instructions for the facilitator, unless otherwise specified.*

***Introductory script:*** *Hi! My name is…, I am from…Thank you for agreeing to participate in this discussion about future HIV prevention products. We will discuss about prevention products that are based on antibodies to prevent HIV infection. We will also discuss other potential options that are based on other approaches like the use of anti-retrovirals which are also used for the treatment of HIV. We want to know about your preferences and considerations for using HIV prevention products.*

We would like to start with understanding the following:

1. What are the HIV prevention methods that you are currently aware of? Do you currently use any of these to help prevent HIV?

- If yes, what is your preferred method of prevention? Why?
- If not, what are some factors that deter you from using them?

1. Before coming to this discussion, have you ever heard anything about broadly neutralising antibodies (bNAbs)?

If yes, tell me what you have heard about this product?

*Probes:*

*From where did you get this information? [Probes: Peers, NGOs/CBOs, social media]*

*To the facilitator:*

*If participants had never heard about bNAbs before (or even if some of the participants have heard of it before), provide a brief (and standard) introduction to bNAbs to ALL participants as follows and provide clarifications if needed.*

- Antibodies are proteins generated by the immune system. They are one of the primary ways the body defends itself against disease. In recent times, antibodies have proved to be one of the most powerful tools to treat untreatable diseases like cancer, autoimmune disorders and also infectious disease like rabies and RSV.
- Antibodies are also being developed for treatment and prevention of a wide range of viral diseases including Zika, dengue, Ebola, influenza, HIV and the newly identified coronavirus, SARS-CoV-2,
- Broadly neutralising antibodies (bNAbs) are antibodies (that neutralize multiple strains of HIV, thereby enabling broad protection against this rapidly mutating virus. These are now being developed as a tool for HIV prevention.
- Over 200 different bNAbs to HIV have been isolated to date and many of these have been tested in animals and clinic for safety and efficacy.
- The bNAb that is the farthest along the development pathway, is currently being tested in clinical trials across sub-Saharan Africa and in the Americas and results are expected in late 2020s;
- Another potential bNAb product is also under development and is expected to go into clinical trials across sub-Saharan Africa and India in a few years.

*The subsequent discussion will be conducted sequentially in two parts as the following:*

1. *Preference elicitation questions (participant-generated, and pre-determined)*
2. *Ranking pre-determined product profiles (four choices)*

**A. PREFERENCE ELICITATION QUESTIONS**

***Discussion on participant-generated attributes/levels first***

1. What are all the things that you would consider before using a bNAb product? Especially, what characteristics or features would you be considering in making a decision whether or not to use it?

- *Ask appropriate follow-up questions and probe as needed to help the participants to come up with individual characteristics/features/attributes.*
- *In general, for each suggestion, ask:*
  - Why do they think that attribute is important?
  - Whether that attribute will be more important for only certain subgroups of people (e.g., if cost is mentioned as an issue, explore whether it will be an issue for all, what about those in sex work, etc.)
- *For each feature/ attribute, probe for the possible “leveIs”/ options. If the attribute matches with the pre-determined attributes in the table below, then after the participants come up with their own levels/options, probe for the left out levels/options.*

*You may refer to Table A below for attributes/levels.*

***Discussion on pre-determined attributes/levels***

*If some of the pre-identified characteristics did not come up in the open discussions above, then specifically ask about the following attributes as provided in the table below. Also probe whether this characteristic will be more important or not at all important for some subgroups (age, identity, sex work status, etc.) and explore why the participants think so?*

*For each characteristic, provide two or three options (“levels”) and assess their reactions.*

Table A: List of attributes/variables that should be probed:

| **Attributes** | **Levels - Acceptable and preferred** |
| --- | --- |
| 1. **Minimum acceptable efficacy** | **Ranges (>90%, 90-70%, 70-50%, 30-50%)** |
| - What according to you should be the minimum ability of the bNAbs to prevent getting infected with HIV? [*After response*] What made you to say so? - Would it be acceptable to you if the range is _____ (*go through the above options*)? - Why/why not? | |
| 1. **Frequency of administration** | **1, 2, 3, 6 months** |
| - What would be the preferred frequency to receive bNAbs? Why? - If bNAbs were provided at ____ month intervals (*go through options*), would that be acceptable? | |
| 1. **Administration**   **Number of injections per visit**  **Volume of each injection**  **Site of injection** | **Injections**  **1, 2, 3**  **1 mL, 2 mL, 3 mL**  **Arm, thigh, butt** |
| - How desirable would it be if bNAbs are provided to you as an injection? Why? - How many injections per visit are you open to receiving? - Would it be acceptable to you if you were given ______ (run through the options) injections per visit? Why/why not? - What would be the acceptable injection volume? - Would fewer number of injections with larger volume be preferred or more number of injections of smaller volume be more acceptable? - Where in your body are you most comfortable to take the injection(s)? Why? - Would you be agreeable if it is given in ____ (*run through the options*)? Why/why not? - Would it be acceptable if bNAbs are provided as an IV – i.e., injected directly into blood vessels? | |
| 1. **Place of Delivery** | **Community setting (NGO/CBO), ICTC/ ART site, Clinic, hospital** |
| - Where would be the preferred location to receive the bNAb product? Why?   - To receive the bNAbs, if it is required to travel to a health facility (ICTC/ART site/clinic/hospital) 4 times a year (every 3 months), would that be feasible?   - *Yes/No*   - *If No, explore what would be the key obstacles* - If it were possible to deliver bNAbs in a community setting (by a trained volunteer or worker belonging to NGO/CBO) would that be preferable instead of going to a health facility for the administration?   - Yes/No   - Why or why not? - If it were possible to give yourself an injection at home, would that be preferable to administration at a health facility or by a community worker?   - Yes/No   - Why or why not?   - If the product would need to be stored in a refrigerator, would that be an issue?   - What home support would be desired to support self-administration? | |
| 1. **Co-administration** | **Yes/No** |
| - Would it be helpful for bNAbs to be provided with other health services? - If so, which services?   - Contraceptives     - (*If contraceptives, explore preferred mode of birth control*)   - Opioid substitution treatment [OST] services [*Facilitator to explain if needed*]   - STD treatment   - Other? | |
| For HIV prevention, would a non-ARV based prevention product be preferable? Why/why not? | |
| 1. **Cost considerations** | |
| - Do you pay for HIV prevention services out-of-pocket, or do you receive support for prevention-related expenses? - What is the maximum you would be able to pay monthly/annually out-of-pocket prevention option? - Would you be willing to pay additional for a product that:   - Requires less frequent dosing?   - Makes injections less painful?   - Reduces the number of injections required?   - Enables self-administration at home? | |
| 1. **Individual Considerations** | |
| Are there any individual considerations you might have in using this product?  *Probes:*   - *Side-effects* - *Adherence* - *Concurrent use of other products (feminizing hormones/drug use/OST/etc).* | |
| 1. **Social Concerns** | |
| Are there any social concerns you might have while using this product?  *Probes:*   - *Partner reactions/concerns* - *Peer reactions/concerns* - *Family reactions/concerns* - *Stigma/discrimination (related to product use and accessing services)* | |
| 1. **Change in risk behaviour** | |
| Do you think the use of this bNAb product would result in any change in sexual or drug injecting behaviours?  *Probes:*   - How much might the risk-taking actions (needle/syringe sharing for PWID, unprotected sex (anal/vaginal) increase or decrease? (A little? A lot? Not at all?) | |

1. **RANKING PRE-DETERMINED PRODUCT PROFILES (BUNDLES)**
2. Imagine that you have been given a choice of four different products with separate bundle of characteristics.

*Show the bundles – A, B, C and D through a mixture of visual methods and IEC tools, to make the attributes and differences as tangible as possible.*

Table B:

| **Attributes** | **Product A** | **Product B** | **Product C** | **Product D** |
| --- | --- | --- | --- | --- |
| **Frequency** | One pill daily | Lead-in daily oral pills for 5 weeks initially, followed by an injection every 2 month | 2 injections every 3 months | 1 implant annually |
| **Formulation** | Pill | Injection | Injection | Implant |
| **Mode of administration** | Self-administered | Health care worker | Health care worker | Health care worker |
| **Site of administration** | Mouth (oral) | Buttocks (in the muscle) | Upper arm (under the skin) | Upper arm (under the skin) |
| **Most common side effects** | Nausea, headache | Short-term injection site pain | Short-term injection site pain | Short-term injection site pain |
| **ARV based product** | Yes | Yes | No | Yes |

**Which one will be your most preferred? Which one will you prefer least? Between the remaining two, if you had to choose one of those two then which one would you choose?**

*Explore – why or why not?*

*This will be done for each of the individuals within the group and ‘why/why not’ will be asked to each of the participants briefly.*
